# Genetic variants of PDGF signaling pathway genes predict cutaneous melanoma survival

**DOI:** 10.18632/oncotarget.20245

**Published:** 2017-08-14

**Authors:** Hong Li, Yanru Wang, Hongliang Liu, Qiong Shi, Hongyu Li, Wenting Wu, Dakai Zhu, Christopher I. Amos, Shenying Fang, Jeffrey E. Lee, Yi Li, Jiali Han, Qingyi Wei

**Affiliations:** ^1^ Department of Clinical Laboratory, The Fourth Hospital of Hebei Medical University, Shijiazhuang, Hebei 050011, China; ^2^ Duke Cancer Institute, Duke Cancer Institute, Duke University Medical Center, Durham, NC 27710, USA; ^3^ Department of Medicine, Duke University School of Medicine, Durham, NC 27710, USA; ^4^ Department of Epidemiology, Fairbanks School of Public Health, Indiana University Melvin and Bren Simon Cancer Center, Indiana University, Indianapolis, IN 46202, USA; ^5^ Community and Family Medicine, Geisel School of Medicine, Dartmouth College, Hanover, NH 03755, USA; ^6^ Department of Surgical Oncology, The University of Texas M.D. Anderson Cancer Center, Houston, TX 77030, USA; ^7^ Department of Biostatistics, University of Michigan, Ann Arbor, MI 48109, USA; ^8^ Department of Population Health Sciences, Duke University School of Medicine, Durham, NC 27710, USA

**Keywords:** cutaneous melanoma, PDGF signaling pathway, single nucleotide polymorphisms, Cox regression

## Abstract

To investigate whether genetic variants of platelet-derived growth factor (PDGF) signaling pathway genes are associated with survival of cutaneous melanoma (CM) patients, we assessed associations of single-nucleotide polymorphisms in PDGF pathway with melanoma-specific survival in 858 CM patients of M.D. Anderson Cancer Center (MDACC). Additional data of 409 cases from Harvard University were also included for further analysis. We identified 13 SNPs in four genes (*COL6A3*, *NCK2*, *COL5A1* and *PRKCD*) with a nominal *P* < 0.05 and false discovery rate (FDR) < 0.2 in MDACC dataset. Based on linkage disequilibrium, functional prediction and minor allele frequency, a representative SNP in each gene was selected. In the meta-analysis using MDACC and Harvard datasets, there were two SNPs associated with poor survival of CM patients: rs6707820 C>T in *NCK2* (HR = 1.87, 95% CI = 1.35-2.59, *P*_meta_ *=* 1.53E-5); and rs2306574 T>C in *PRKCD* (HR = 1.73, 95% CI = 1.33-2.24, *P*_meta_ *=* 4.56E-6). Moreover, CM patients in MDACC with combined risk genotypes of these two loci had markedly poorer survival (HR = 2.47, 95% CI = 1.58-3.84, *P* < 0.001). Genetic variants of rs6707820 C>T in *NCK2* and rs2306574 T>C in *PRKCD* of the PDGF signaling pathway may be biomarkers for melanoma survival.

## INTRODUCTION

Cutaneous melanoma (CM) is the most aggressive form of skin cancer. Its incidence rates continuously increased in white men (2.1% per year) and women (2.4% per year) between 1999 and 2008 in the United States [1]. It is predicted that there will be 76,380 new cases and 10,130 deaths of CM in the United States in 2016 [2]. To date, tumor Breslow thickness, tumor stage, ulceration and mitotic rate remain the most important prognostic factors for CM patients [3]. In general, CM patients with thinner tumors have a longer survival than those with thicker tumors, and currently all patients with microscopic nodal metastases, regardless of the extent of tumor burden, are classified as stage III, including micrometastases detected by immunohistochemistry [3]. However, these methods are not sufficient to accurately discriminate CM patients for personalized clinical assessment or prediction of their survival, and additional effective clinical or molecular characterization of CM patients with more accurate prognostic potential for personalized health care is needed [4, 5]. There is growing evidence for the role of genetic (germline) variants in CM prognosis [5], and genetic variant discovery may also provide clues about the mechanisms underlying melanocyte carcinogenesis and CM progression, leading to improved prediction of CM prognosis.

A number of investigations have indicated that the platelet-derived growth factor (PDGF) family members are highly expressed in some tumors, including CM, and play important roles in stromal fibroblast recruitment [6, 7]. PDGF is one of the most extensively studied regulators of mesenchymal cell proliferation and migration [8] and an important molecule involved in the control and modulation of the interactions between pericytes and endothelial cells [9]. PDGF influences biologic function through the PI3/MAP-kinase pathway [10], results in fibroblast glycosaminoglycan-stimulating activity, and induces hyaluronan (HA) synthesis [11]. PDGFR signaling is also involved in reciprocal interactions between tumor cells and stroma, thus mediating angiogenesis [12–14]. The PDGF family consists of four polypeptide chains (PDGF-A –B –C and –D) that dimerize to form five biologically active growth factors (i.e., PDGF-AA –AB –BB –CC and –DD). While the PDGFα receptor binds to PDGF-A –B and –C chains, the PDGFβ receptor binds to PDGF-B and PDGF-D chains, and the binding receptors of PDGF isoforms stimulate mesenchymal origin cells to proliferate, migrate and survive [15, 16]. The PDGF-A has been documented to stimulate tumor growth in an autocrine fashion [17–19], while the PDGF-B may be implicated in stroma recruitment and can facilitate tumor growth through its paracrine effects on stromal cells [20]. Likewise, The PDGF-C maintains growth-promoting tumor microenvironment [18, 21], especially in CM [22], and the PDGF-D accelerates tumor growth through the activation of adjacent stromal cells [23].

Hence, it is likely that the PDGF signaling pathway is important to some cancers and to CM in particular. This motivated us to investigate whether genetic variants of the PDGF signaling pathway genes are associated with survival of CM patients, using published genome-wide association study (GWAS) datasets [24, 25].

## RESULTS

### Patient characteristics in The University of Texas MD Anderson Cancer Center (MDACC) and Harvard University GWASs

The MDACC GWAS included 858 melanoma patients with complete information in clinicopathological features [24]. The patients were aged between 17 and 94 years at diagnosis (52.4 ± 14.4 years), and there were more men (496, 57.8%) than women (362, 42.2%). More patients in stages I/II (709, 82.6%) than those in stages III/IV (149, 17.4%) at presentation. After the initial GWAS, 95 patients died of CM. In univariate analyses, six variables, including age, sex, tumor stage, Breslow thickness, ulceration and mitotic rate, were marginally or significantly associated with melanoma-specific survival (MSS). In multivariate analyses, only tumor stage, Breslow thickness, and ulceration were found to be significantly associated with MSS. (Supplementary Table 1). There were 409 cases available for survival analysis in the Harvard GWAS [25]. Eligible cases were between 34 to 87 years of age at diagnosis (61.1 ± 10.8 years). A total of 48 CM-specific deaths were observed in the Harvard dataset, and only age was significantly associated with MSS in the univariate analysis (Supplementary Table 1). Besides follow-up information and genotype data, only age and sex were available in the Harvard study. Therefore, subsequent stratification or subgroup analyses by clinical variables and multivariate analyses with adjustment for clinical variables were performed using the MDACC dataset only.

### Multivariate analysis of single nucleotide polymorphisms (SNPs) in MDACC patients

**Figure 1:**
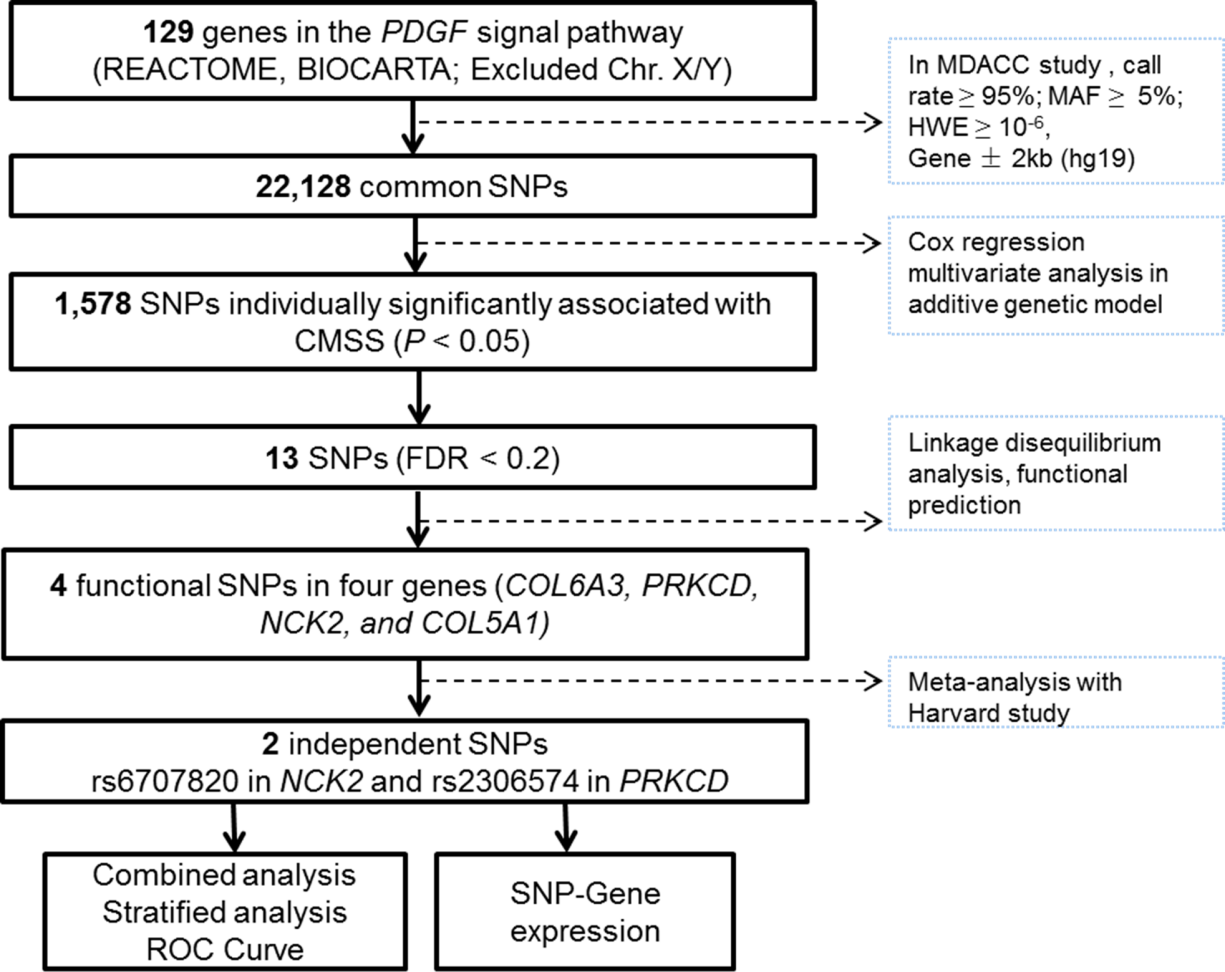
Study workflow: we firstly chose 129 PDGF-related genes from the databases integrated in MSigDB (http://software.broadinstitute.org/gsea/msigdb) and included a total of 22,128 genotyped or imputed SNPs that were located within 2-kb upstream and downstream of those genes in the MDACC dataset; 13 SNPs were found significantly associated with FDR < 0.2 and four intendent SNPs were further chosen to be replicated in the Harvard study; there were two SNPs with consistent, by non-significant, associations with melanoma-specific survival in the Harvard study, and further functional analyses were performed

The 129 autosome genes in the PDGF signaling pathway were extracted from the Molecular Signatures Database (http://software.broadinstitute.org/gsea/msigdb/search.jsp). A total of 22,128 SNPs located within 2-kb upstream and downstream of the 129 genes were extracted from the GWAS datasets (Figure 1).

In multivariate Cox regression analysis with adjustments for age, sex, Breslow thickness, tumor stage, ulceration and mitotic rate, we found that 1,578 SNPs were significantly associated with MSS at *P* < 0.05 in a signal-locus analysis with an additive genetic model. Among these 13 SNPs with FDR < 0.2 in four genes were considered significant after multiple test correction Supplementary Figure 1A). Potential functionality of the 13 SNPs was predicted by using SNPinfo and RegulomeDB (Supplementary Table 2). According to functional prediction, minor allele frequency (MAF), and linkage disequilibrium, we selected a representative SNP for each of the four genes as potentially functional SNPs for further analyses (*NCK2* rs6707820 C>T, *PRKCD* rs2306574 T>C, *COL5A1* rs13301426 C>T and *COL6A3* rs2645768 A>C, Supplementary Figure 1B). These four putative functional SNPs together with clinical prognostic variables were selected into a multivariate stepwise Cox model. As a result, four independent functional SNPs remained significantly associated with MSS at *P* ≤ 0.05 (Supplementary Table 3).

### Meta-analysis of the results from MDACC and Harvard studies

We assessed the associations of the four identified SNPs in the Harvard study with adjustment for age and sex. Two SNPs (*NCK2* rs6707820 C>T and *PRKCD* rs2306574 T>C) showed consistent, but non-significant, associations with MSS in this independent study. In the meta-analysis of MDACC and Harvard studies, the two SNPs remained significantly associated with MSS (HR = 1.87, 95% CI = 1.35-2.59, *P*_meta_ = 1.53E-5; HR = 1.73, 95% CI = 1.33-2.24, *P*_meta_ = 4.56E-6, respectively.) (Table 1).

**Table 1:** Meta-analysis of four independent SNPs in MDACC and Harvard databases

SNP	Allele^a^	Gene	MDACC (n=858)^b^		Harvard (n=409)^c^		Meta-analysis^d^			
			HR (95%CI)	*P*	HR (95%CI)	*P*	*P*_het_	I^2^(%)	HR (95%CI)	*P*
rs6707820	C>T	*NCK2*	2.19 (1.48-3.25)	9.53E-05	1.34 (0.76-2.37)	0.310	0.164	48.4	1.87 (1.35-2.59)	1.53E-05
rs2306574	T>C	*PRKCD*	1.92 (1.40-2.64)	6.46E-05	1.31 (0.86-2.19)	0.181	0.242	27.1	1.73 (1.33-2.24)	4.56E-06
rs2645768	A>C	*COL6A3*	1.91 (1.41-2.58)	2.40E-05	0.65 (0.37-1.14)	0.131	0.001	90.9	1.14 (0.40-3.29)	0.801
rs13301426	C>T	*COL5A1*	2.36 (1.53-3.64)	9.54E-05	1.05 (0.52-2.14)	0.903	0.056	72.7	1.66 (0.75-3.64)	0.210

MDACC: The University of Texas M.D. Anderson Cancer Center; HR: hazards ratio; CI: confidence interval; *P*_*het*_*: P*-value for heterogeneity test, SNP: single nucleotide polymorphism.

^a^Reference allele/effect allele.

^b^Adjusted for age, sex, Breslow’s tumor thickness, regional/distant metastasis, ulceration and mitotic rate.

^c^Adjusted for age and sex.

^d^If *P*_het_ <0.10 or I^2^>50%, random-effects model was selected; otherwise fixed-effect model was selected.

Although no melanoma-specific survival data available in the TCGA database, we also explored the correlations of the four identified SNPs with the overall survival of 287 melanoma patients in the TCGA database with available information about age, sex and stage. However, no significant result was found for these four SNPs (Supplementary Table 4).

### Genetic variants in the PDGF signaling pathway genes as independent survival predictor in MDACC patients

**Figure 2:**
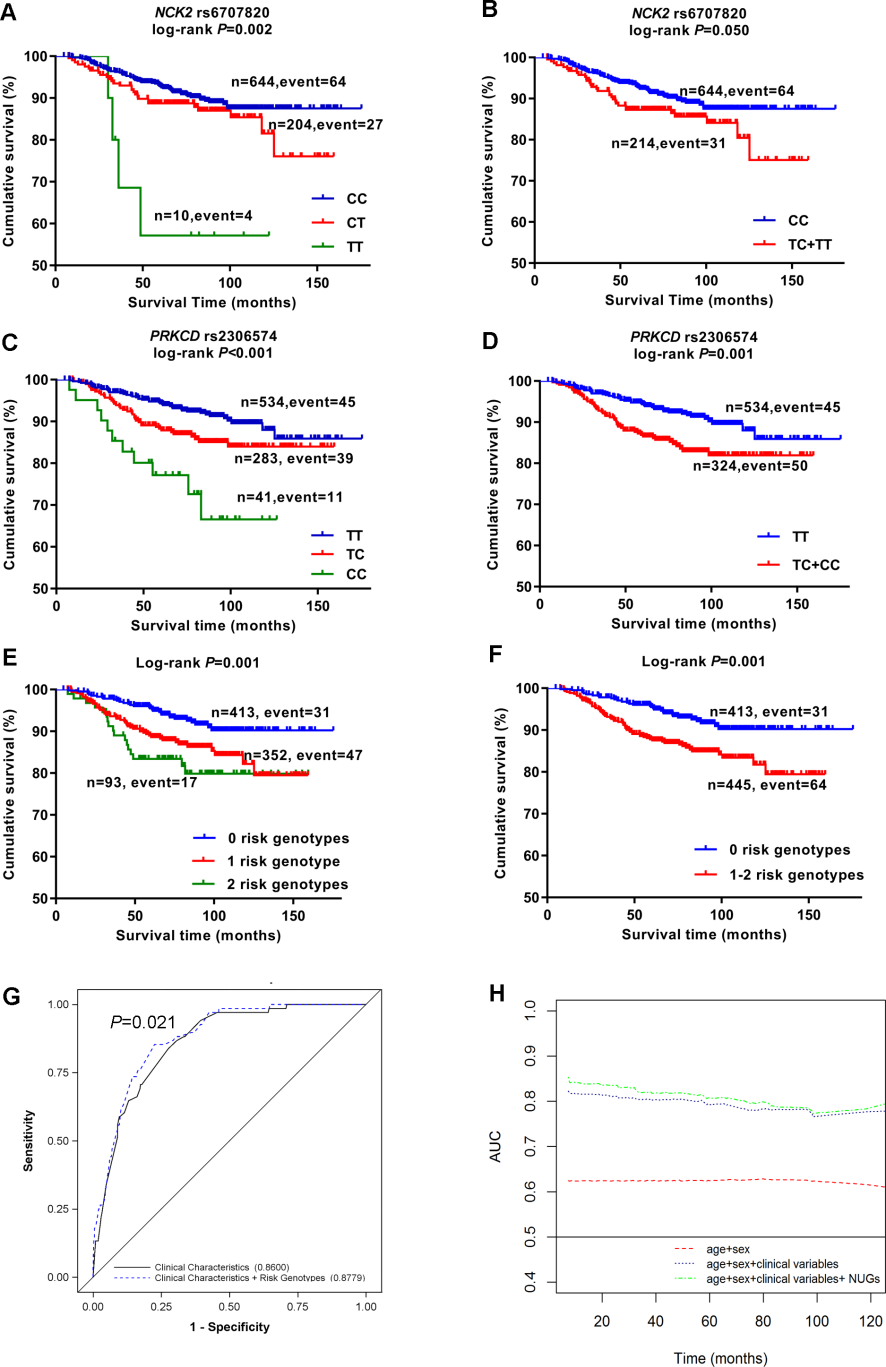
Kaplan-Meier survival curves, receiver operating characteristic (ROC) curves and time-dependent AUC for prediction of melanoma-specific survival Cutaneous melanoma-specific survival stratified by genotypes of rs6707820 in *NCK2* **(A**, **B)** and rs2306574 in *PRKCD* **(C**, **D)** in additive and dominant model. Kaplan-Meier estimates of melanoma-specific survival by numbers of unfavorable genotypes **(E)**, dichotomized subgroups by the numbers of unfavorable genotypes **(F)**. ROC curves for five-year melanoma-specific survival prediction **(G)**, time-dependent AUC based on age, sex, regional/distant metastasis, Breslow thickness, ulceration, mitotic rate, and number of unfavorable genotypes **(H)**.

As shown in Table 2, the effects of that two SNPs remained significant in the MDACC dataset after adjustment for age, sex, Breslow thickness, regional/distant metastasis, ulceration, mitotic rate of tumors [rs6707820 CT+TT vs. CC: adjusted hazards ratio (adjHR) = 2.05, 95% confident interval (CI) = 1.31-3.22, *P* = 0.002; rs2306574 CT+CC vs. TT: adjHR = 1.97, 95% CI = 1.30-2.99, *P* = 0.001]. As shown in Figure 2A–2D, these two SNPs had a significant association with poor MSS in both additive and dominant model (*P*_additive_= 0.002 and *P*_dominant_= 0.050 for SNP rs6707820; *P*_additive_ < 0.001, *P*_dominant_= 0.001 for rs2306574).

**Table 2:** Associations between MSS of CM patients and selected SNPs of the PDGF pathway in the MDACC study

Genotype	Frequency	Death (%)	Univariate analysis		Multivariate analysis*	
			HR (95% CI)	*P*	HR (95% CI)	*P*
***NCK2***						
rs6707820 C>T (genotyped)						
CC	644	64 (9.9)	1.00		1.00	
CT	204	27 (13.2)	1.39 (0.89-2.18)	0.153	1.87 (1.16-2.99)	0.010
TT	10	4 (40.0)	4.99 (1.82-13.71)	0.002	6.58 (2.29-18.90)	0.001
Trend test				0.012		<0.001
CT+TT	214	31 (14.5)	1.53 (1.00-2.35)	0.052	2.05 (1.31-3.22)	0.002
***PRKCD***						
rs2306574 T>C (imputed)						
TT	534	45 (8.4)	1.00		1.00	
CT	283	39 (13.8)	1.70 (1.11-2.61)	0.016	1.68 (1.07-2.63)	0.023
CC	41	11 (26.8)	3.92 (2.03-7.59)	<0.001	4.45 (2.26-8.74)	<0.001
Trend test				<0.001		<0.001
CT+CC	324	50 (15.4)	1.94 (1.30-2.90)	0.001	1.97 (1.30-2.99)	0.001

MSS: melanoma-specific survival; CM: cutaneous melanoma; SNP: single nucleotide polymorphism; MDACC: The University of Texas M.D. Anderson cancer center; HR: hazards ratio; CI: confidence interval.

* Adjusted for age, sex, Breslow thickness, regional/distant metastasis, ulceration, mitotic rate of tumors.

### Survival of MDACC patients with unfavorable genotypes of the two SNPs

We combined risk genotypes of *NCK2* rs6707820 C>T and *PRKCD* rs2306574 T>C into a composite count variable as the number of unfavorable genotypes (NUGs) in the MDACC study. All patients were separated into three groups with 0, 1, and 2 NUGs, which had 413, 352, and 93 patients, respectively. The per-unit increase in NUG was associated with an increased risk of death (*P*_trend_ < 0.001, Table 3). We then divided all patients into a low-risk group with 0 NUGs and a high-risk group with 1-2 NUGs. The high-risk group had a two-fold increased risk of death (adjHR = 2.47, 95% CI = 1.58-3.84, *P* < 0.001), compared with the low risk group with adjustment for clinical covariates. To illustrate these results, Kaplan-Meier curves are shown in Figure 2E–2F.

**Table 3:** HRs for associations between NUGs and MSS in CM patients of the MDACC study

NUG^a^	Frequency		Univariate analysis		Multivariate analysis^b^	
	All	Death (%)	HR (95% CI)	*P*	HR (95% CI)	*P*
0	413	31 (7.5)	1.00		1.00	
1	352	47 (13.4)	1.92 (1.22-3.02)	0.005	2.30 (1.43-3.68)	0.001
2	93	17 (18.3)	2.62 (1.45-4.73)	0.001	3.02 (1.66-5.51)	<0.001
Trend test				<0.001		<0.001
0	413	31 (7.5)	1.00		1.00	
1-2	445	64 (14.4)	2.07 (1.35-3.17)	0.001	2.47 (1.58-3.84)	<0.001

NUG: number of unfavorable genotype; MSS: melanoma-specific survival; CM: cutaneous melanoma; SNP:single nucleotide polymorphism; MDACC: The University of Texas M.D. Anderson Cancer Center; HR: hazards ratio; CI: confidence interval.

^a^Number of risk genotypes were derived from rs2306574 CC+CT and rs6707820 TT+CT.

^b^Multivariate Cox regression analyses were adjusted for age, sex, Breslow thickness, regional/distant metastasis, ulceration, and mitotic rate of tumor.

### Stratified analyses for NUGs in MDACC patients

Compared with the low-risk group, the high-risk group showed a remarkably increased risk of death in patients who had Breslow thickness > 1 mm, Mitotic rate > 1, ulceration and regional/distant metastasis. However, there was no evidence for a multiplicative interaction of risk genotypes with any of clinical variables (all *P*_interaction_ > 0.050, Table 4).

**Table 4:** Associations in stratified analysis of MSS and NUG across genes in the MDACC study

Characteristics	0 NUGs^a^		1-2 NUGs^a^		Multivariate analysis^b^		*P*_interaction_^c^
	All	Death (%)	All	Death (%)	HR (95% CI)	*P*	
Age							0.154
≤50	183	7 (3.8)	188	24 (12.8)	4.25 (1.73-10.49)	0.002	
>50	230	24 (10.4)	257	40 (15.6)	1.86 (1.10-3.16)	0.021	
Sex							0.874
Female	180	9 (5.0)	182	17 (9.3)	2.67 (1.13-6.30)	0.025	
Male	233	22 (9.4)	263	47 (17.9)	2.37 (1.40-4.00)	0.001	
Regional/distant metastasis							0.104
No	342	17 (5.0)	367	34 (9.3)	1.84 (1.01-3.34)	0.046	
Yes	71	14 (19.7)	78	30 (38.5)	3.40 (1.72-6.72)	<0.001	
Breslow's tumor thickness (mm)							0.221
≤1	174	3 (1.7)	173	4 (2.3)	0.65 (0.11-3.72)	0.626	
1-2	140	9 (6.4)	136	17 (12.5)	2.14 (0.93-4.91)	0.074	
2-4	64	13 (20.3)	103	28 (27.2)	1.72 (0.87-3.42)	0.120	
>4	35	6 (17.1)	33	15 (45.5)	3.36 (1.25-9.03)	0.016	
Ulceration							0.068
No	335	18 (5.4)	346	30 (8.7)	1.66 (0.92-2.99)	0.090	
Yes	70	13 (18.6)	85	30 (35.3)	3.79 (1.88-7.62)	<0.001	
Missing	22						
Mitotic rate (mitoses/mm^2^)							0.398
<1	132	3 (2.3)	143	6 (4.2)	1.68 (0.40-7.08)	0.481	
≥1	281	28 (10.0)	302	58 (19.2)	2.10 (1.34-3.30)	0.001	

MSS: melanoma-specific survival; NUG, number of risk genotype; MDACC: The University of Texas M.D. Anderson Cancer Center; HR: hazards ratio; CI: confidence interval.

^a^number of risk genotypes were derived from rs2306574 CC+CT and rs6707820 TT+CT.

^b^Multivariate Cox regression analyses were adjusted for age, sex, Breslow thickness, distant/regional metastasis, ulceration of tumor and tumor cell mitotic rate, where appropriate.

^c^*P*-value for multiplicative interaction.

### Receiver operating characteristic curve and time dependent area under the curve (AUC) in MDACC patients

We further evaluated the NUG for its potential to predict CM prognosis by receiver operating characteristic curve (Figure 2G). The AUC of the 5-year MSS significantly increased from 86.0% to 87.8% (*P* = 0.021, DeLong’s test). In the time-dependent AUC, the plot indicated an improved prediction performance with the adding of NUGs to the multivariate model between the beginning and the remaining of the follow-up over times (Figure 2H).

### *In silico* functional analyses

**Figure 3:**
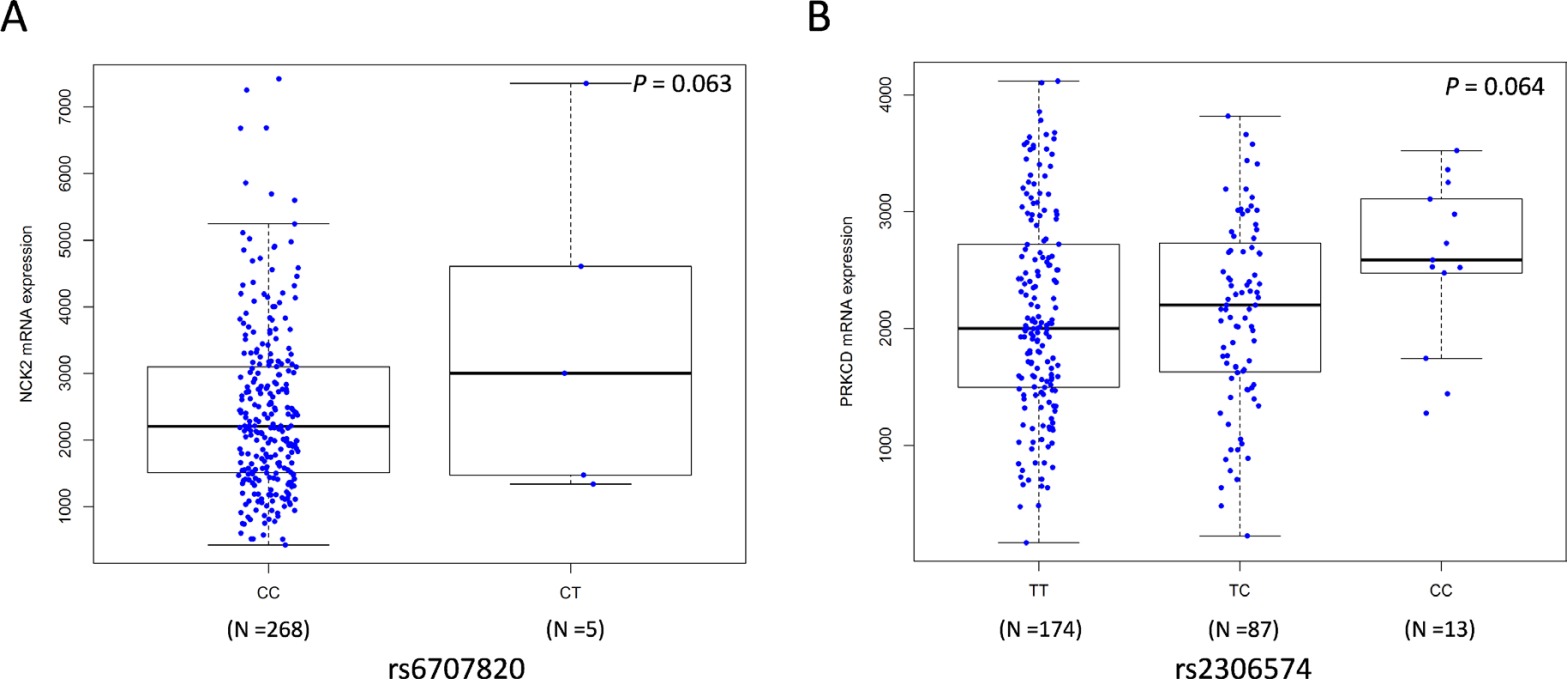
Correlations between SNPs and mRNA expression levels of *NCK2* and *PRKCD* Analyses of mRNA expression levels by genotypes of *NCK2* rs6707820 **(A)** and *PRKCD* rs2306574 **(B)** in an additive genetic model using the data from 284 metastatic melanoma tissues in the TCGA database. The Y-axis shows the normalized mRNA expression level. The boxes represent the median (black middle line) limited by the first (Q1) and third (Q3) quartiles. The whiskers are the upper and lower adjacent values, which indicate the value of Q3+1.5(Q3−Q1) or the maximum value [if it is less than Q3+1.5(Q3−Q1)], and the value of Q1−1.5 (Q3−Q1) or the minimum value [if it is greater than Q3+1.5(Q3−Q1)], respectively.

We also performed the expression quantitative trait loci (eQTL) analysis by using mRNA expression data of 284 metastatic melanoma tissues from the TCGA database [26]. We found that the rs6707820 T allele and rs2306574 C allele was correlated with increased mRNA expression levels of *NCK2* and *PRKCD*, respectively (*P*_additive_ = 0.063 and *P*_*additive*_ = 0.064, respectively Figure 3A–3B). The Genotype-Tissue Expression (GTEx) project collected transcriptome data in a wide variety of tissue types from post-mortem donors(http://www.gtexportal.org) [27]. In GTEx, we found that the rs2306574 C allele was associated with an increased mRNA expression level of *PRKCD* in data from the sun-exposed skin (*P* < 0.001, Supplementary Figure 3A), which is consistent with that in metastatic tissues. However, the rs6707820 T allele showed a significant correlation with lower mRNA expression levels of *NCK2* (*P*_additive_ = 0.036, Supplementary Figure 3B) in lymphoblastoid cell-lines from 373 Europeans in the 1000 Genomes Project [28].

We further investigated the difference in the expression levels of the two genes between CM and normal skin tissues in the publicly available Oncomine Compendium of Expression Array Data (https://www.oncomine.org/) [29]. The *PRKCD* expression levels in tumor tissue were higher than that in normal tissue in the Talantov study that included 45 CM tumor tissues and seven normal skins (*P* < 0.001, Supplementary Figure 3C). The *NCK2* also showed a higher expression level in CM tumor tissue in both Talantov (*P* < 0.001) and Riker studies that included 14 CM tumor tissues and four normal skins (*P* = 0.023) (Supplementary Figure 3D–3E).

## DISCUSSION

In the present study, we found that genetic variants of two *PDGF* pathway genes, namely *PRKCD* rs2306574 T>C and *NCK2* rs6707820 C>T, were likely to independently or jointly modulate survival of CM patients. We demonstrated that combination of the number of *PRKCD* and *NCK2* risk genotypes with other known clinicopathologic variables in the same multivariable model significantly improved the prediction accuracy of MSS in CM patients.

*PRKCD* located on chromosome 3p21.1 belongs a novel class serine/threonine kinase of the protein kinase C family [30], and it is also regulated by phosphorylation on various serine/threonine (e.g. T50, T141, S304, T451, T505, S506, T507, S643, and S664) and tyrosine residues including Y311 [31, 32]. PRKCD suppresses immunoresponsiveness and inhibits the proliferation of B-lymphocytes [33], and *PRKCD* has an indispensable function in negatively regulating B-cell proliferation, which is particularly important for the establishment of B-cell tolerance [34]. *PRKCD* appears to have dual functions in carcinogenesis not specific for a particular tumor type [35]. For example, *PRKCD* can be a suppressor in a few cancer types [33, 36], but over-expression of *PRKCD* increased the metastatic potential of murine BL16 mouse melanoma cells [37, 38]. PRKCD is not required for the proliferation or survival of normal cells, but it is required for proliferation of multiple types of cancer cells, both *in vitro* and *in vivo*, including those from cancers of the breast, pancreas and prostate as well as melanoma [39]. By using the TCGA and GTEX data, we also showed that the rs2306574 variant C allele was correlated with increased mRNA expression levels in metastatic melanoma tissues and the sun exposure skin. The present study supports *PRKCD* to be a predictor for survival of CM patients, which is further supported by the high expression levels of *PRKCD* in CM tumor tissues than in the normal skin from the Oncomine gene expression database.

The *NCK2* gene located on chromosome 2q12.2 is involved in signaling pathways mediating proliferation, cytoskeleton organization and integrated stress response [40–42]. NCK proteins belong to the SH2/SH3 adaptor proteins, and there are two known family members including *Nck1*/*Nck*/*Nckα* and *Nck2*/*Nckβ*/*GRB4* (growth factor receptor binding protein 4) [40, 43]. It has been shown that Nck2 is specifically involved in the PDGF-induced membrane ruffling and the formation of lamellipodia. *Nck2* acts either in parallel with or downstream of Rac1, a mediator between PDGFR and the actin cytoskeleton, and that Nck2, but not Nck1, blocks the PDGF-induced actin polymerization and plays a specific role in the PDGFR signaling to the actin cytoskeleton [44]. Other studies have suggested that the middle SH3 domains of Nckα and Nckβ could independently mediate PDGF-BB signaling to promote human dermal fibroblasts (HDFs) migration, and that overexpression of the middle SH3 domain of Nckα or Nckβ alone in HDFs could also block PDGF-BB-induced cell migration [45]. Furthermore, in another investigation Nck2 protein and mRNA levels were increased in human metastatic melanoma cells compared with human primary melanoma cells that rarely metastasized. Nck2 promoted cell proliferation, migration and invasion in human melanoma cells; increased Nck2 expression in human primary melanoma cells promoted the melanoma-derived tumor growth rate; and Nck2 promoted phosphorylation of proteins on tyrosine and down-regulation of cell surface adhesion proteins in human primary melanoma cells [41].

Finally, in other studies up-regulation of NCK2 was present in melanoma tumor samples from metastasis compared to nevocellular nevus lesions by semi-quantitative RT–PCR and custom array analysis, suggesting a role of NCK2 in melanocytic tumor progression [46]. Consistent with this observation, the Oncomine cancer microarray data (https://www.oncomine.org/) also suggested that Nck2 was up-regulated in human melanoma in both Talantov and Riker melanoma studies in the Oncomine Compendium [28]. Because *NCK2* is considered an oncogene, its variants may play an important role in CM progression. We also found marginally significant associations between the variant allele of *NCK2* rs6707820 and increased mRNA expression levels of *NCK2* in metastatic melanoma tissues, although inconsistent eQTL results were found in the lymphoblastoid cell lines from normal CEU people, which may due to tissue heterogeneity. Taken together, the variant rs6707820 in the *NCK2* gene may be a predictor for survival in CM patients.

In the present study, we found interesting associations of MSS with some novel genetic variants (i.e. *PRKCD* rs2306574 T>C, *NCK2* rs6707820 C>T *COL5A1*, rs13301426 C>T, and *COL6A3* rs2645768 A>C)*.* These SNPs could modulate MSS independently in MDACC patients, and the combined NUG of these SNPs could better discriminate prognostic groups in multivariate analyses, independent of other clinical characteristics. These findings suggest that PDGF pathway genetic variants might have biological roles in CM progression. However, the present study has some limitations. In the meta-analysis of the selected SNPs from MDACC data and Harvard data, only rs6707820 in *NCK2* and rs2306574 in *PRKCD* remained statistically significant in predicting MSS. Because of unavailable data from the Harvard study, we could only perform multivariate analyses using the MDACC data. Therefore, additional validation is warranted.

## MATERIALS AND METHODS

### Study populations and SNP genotyping

The original MDACC study had 1,803 patients accrued for a hospital-based case-control study of CM, and the characteristics of these patients have also been described previously [47]. The 858 patients included for survival analysis were part of a molecular epidemiology study in which complete information for clinical prognostic variables and questionnaire data were collected. Tissue samples were collected as whole blood and DNA were extracted with various methods (including Gentra, Qiagen and phenol/chloroform). Genotyping was performed by the Illumina HumanOmni-Quad_v1_0_B array and requested from the Database of Genotypes and Phenotypes (accession: phs000187.v1.p1) [48, 49]. Genome-wide imputation was conducted with the MACH software based on the 1000 Genomes Project phase I v2 CEU data [50]. The detailed genotyping information and data quality control can be found in the previously reported GWAS [47].

The Harvard study consisted of two cohorts: NHS (Nurses’ Health Study) and HPFS (Health Professionals Follow-up Study), established respectively in 1976 and 1986 [25]. All patients in both cohorts were diagnosed with histopathologically confirmed invasive CM after baseline until the 2008 follow-up cycle; there were 409 patients eligible for survival analysis. DNA was extracted from the collected whole blood samples and genotyping was performed using the Illumina HumanHap610 array. Genome-wide imputation was also performed using the MACH software based on the 1000 Genomes Project CEU data (phase I v3, March 2012) [50]. All written informed consent was obtained from each participant of the two studies. All methods were performed in accordance with the relevant guidelines and regulations, and the present study followed the study protocols approved by the institutional review board for each of the participating institutions.

### Gene and SNP selection

Based on the databases of BIOCARTA and REACTOME (http://software.broadinstitute.org/gsea/msigdb/search.jsp), we included 129 genes located on autosomes from the PDGF signaling pathway after deleting duplicate genes. The genotyped or imputed SNPs located within 2 kb upstream and downstream of the PDGF pathway genes were extracted from the GWAS datasets with genotyping call rate ≥ 95%, MAF ≥ 5%, Hardy-Weinberg Equilibrium exact *P* value ≥ 10^-6^and imputation r^2^ ≥ 0.8. As a result, there were 22,128 SNPs including 3,315 genotyped SNPs and 18,813 imputed SNPs in MDACC study.

### Statistical methods

MSS was determined from the time of diagnosis until death from CM; individuals who died of causes other than CM were censored. Associations between SNPs and MSS (in an additive model) were assessed by Cox proportional hazards regression analyses performed with GenABEL package of R software [51] with adjustments for age, sex, tumor stage, Breslow thickness, tumor cell mitotic rate, and ulceration of tumor in the MDACC study [3]. The FDR cut-off of 0.2 was applied to limit the probability of false positive findings as abundant of SNPs had been tested. The multivariable stepwise Cox model including four functional SNPs and clinical variables was carried out to choose the independent SNPs. A meta-analysis was used to combine the results from two studies. Kaplan-Meier survival curves and log-rank tests were used to evaluate the effects of genetic variants on the cumulative probability of MSS. The multiplicative interaction between subgroups was assessed with the logistic regression, and the multiplicative interaction was considered significant when *P* < 0.050. Receiver operating characteristic curve was constructed from the logistic regression model, and the NUG was used to assess the classification performance of the model. Statistical significance of the improvement in NUGs after adding an explanatory factor was calculated by the Delong’s test [52]. For more biological relevance of our findings, we searched the Oncomine website database (https://www.oncomine.org/resource/login.html) for studies that provided gene mRNA expression data from melanoma cases. Linear regression analysis was used to test for the trends in the associations between SNPs and corresponding gene expression levels obtained from the 373 lymphoblastoid cell lines from the 1000 Genomes European population and other datasets from GTEx website [27] and the Cancer Genome Atlas (TCGA) database (dbGaP Study Accession: phs000178.v1.p1) [26]. All other analyses were performed using SAS software (Version 9.4; SAS institute, Cary, NC), unless otherwise specified.

## SUPPLEMENTARY MATERIALS FIGURES AND TABLES



## Abbreviations

adjHR: adjusted hazard ratio
AUC: area under the curve
CI: confident interval
CM: Cutaneous melanoma
eQTL: expression quantitative trait loci
FDR: false discovery rate
GTeX: Genotype-Tissue Expression project
GWAS: genome-wide association study
HPFS: Health Professionals Follow-up Study
PDGF: platelet-derived growth factor
MAF: minor allele frequency
MDACC: M.D. Anderson Cancer Center
MSS: melanoma-specific survival
NHS: Nurses’ Health Study
NUGs: number of unfavorable genotypes
SNP: single nucleotide polymorphism
