# Supplementary material for: Genetic variants of PDGF signaling pathway genes predict cutaneous melanoma survival
Source: Oncotarget. 2017 Aug 14;8(43):74595–606. doi: 10.18632/oncotarget.20245 (PMC5650365; doi:10.18632/oncotarget.20245)
Supplement: Supplementary file 1 [file oncotarget-08-74595-s001.pdf]

Genetic variants of PDGF signaling pathway genes predict cutaneous melanoma survival

SUPPLEMENTARY MATERIALS

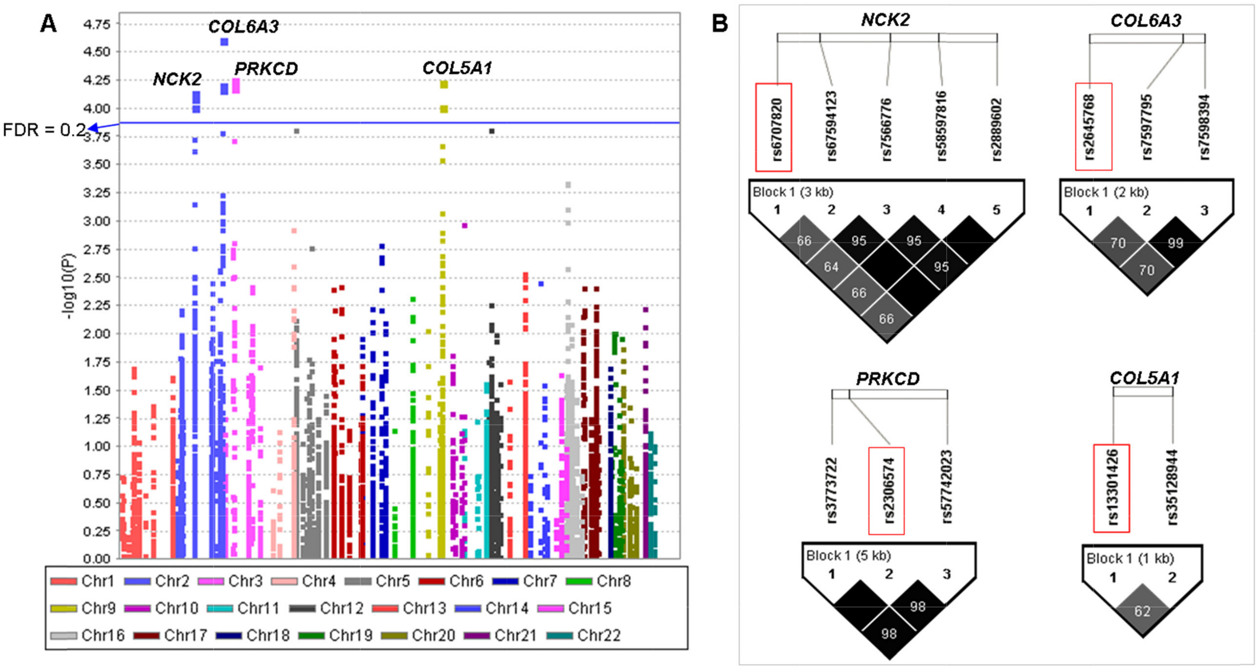

Supplementary Figure 1: Screening for SNPs in the PDGF signaling pathway genes. **(A)** Manhattan plot of associations between 22,128 SNPs in the PDGF Signaling pathway and CMSS. There were 1,578 SNPs with *P*-value < 0.05 and 13 SNPs retained after FDR correction. The blue horizontal line indicates FDR = 0.20. **(B)** Linkage disequilibrium (LD) relationships of the 13 SNPs in four genes using the 1000 Genomes European population, and the independent SNP of each gene was marked in red.

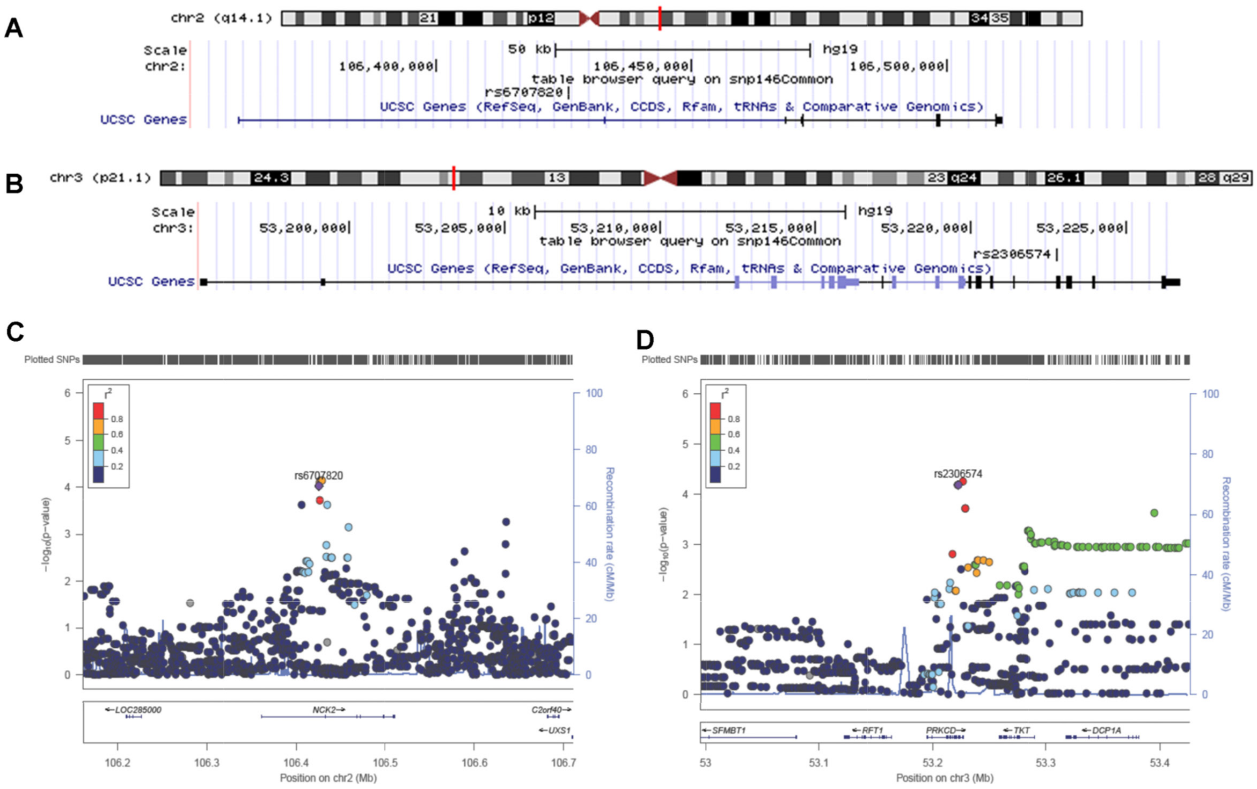

Supplementary Figure 2: Locations of rs6707820 and rs2306574 in gene regions from the UCSC genome browser (A, B) and regional association plots of *NCK2* (C) and *PRKCD* (D). The left-hand Y-axis shows the *P*-value of each SNP, which is plotted as -log10 (*P*) against chromosomal base-pair position. The right-hand Y-axis shows the recombination rate estimated from the HapMap CEU population.

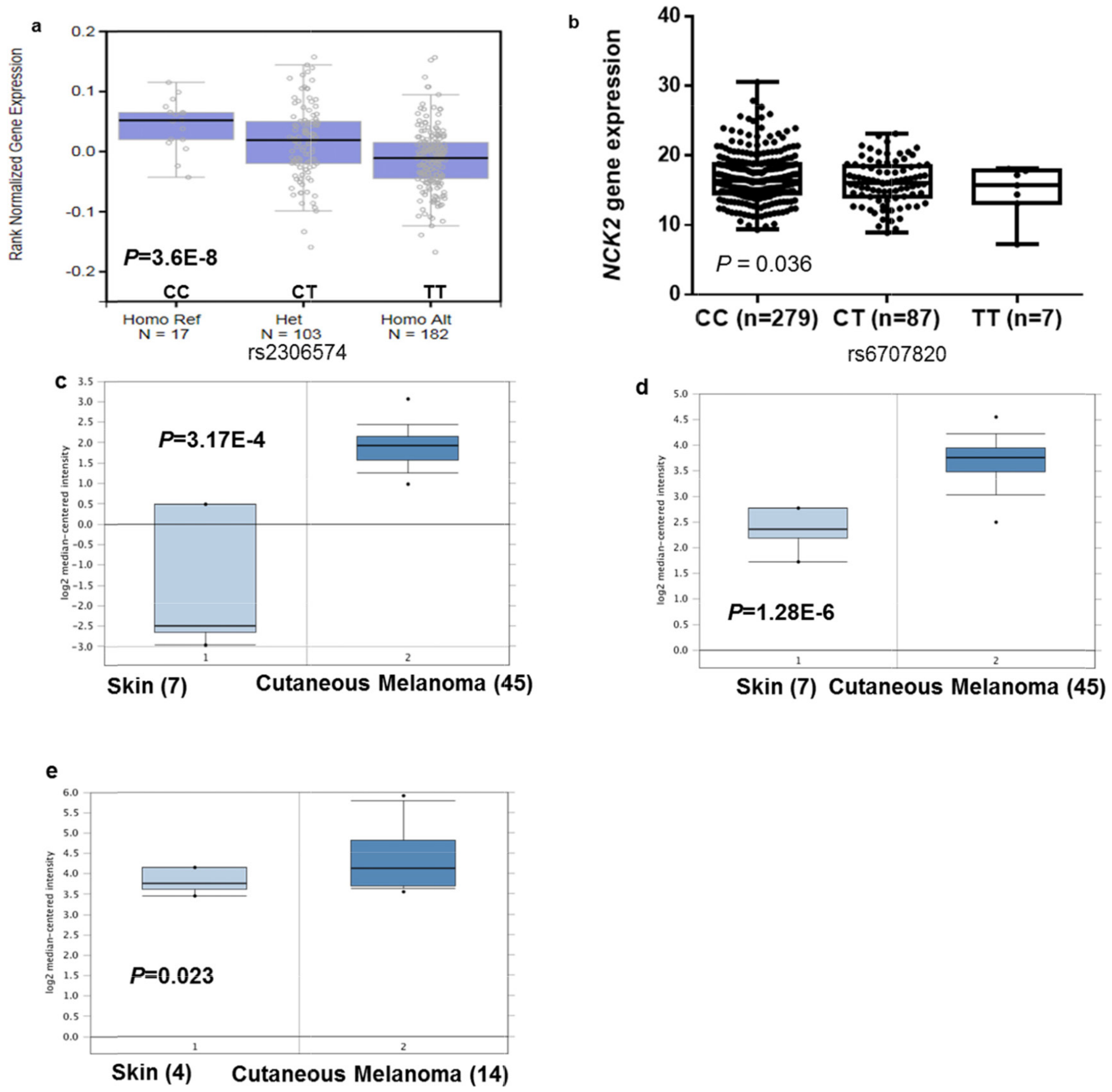

Supplementary Figure 3: (a) eQTL result of *PRKCD* rs2306574 (T>C) genotype in sun-exposed skin from the GTEx project (http://www.gtexportal.org) **(b)** eQTL results of *NCK2* rs6707820 (C>T) genotype in lymphoblastoid cell-lines from 373 Europeans in the 1000 Genomes Project **(c)** *PRKCD* Expression in Talantov Melanoma, **(d)** *NCK2* expression in Talantov Melanoma, **(e)** expression in Riker Melanoma from Oncomine database (https://www.oncomine.org/resource).

Supplementary Table 1: Characteristics of the MDACC and Harvard studies

Parameter	Frequency	Death (%)	Univariate analysis		Multivariate analysis*	
			HR (95% CI)	*P*	HR (95% CI)	*P*
**MDACC**	858	95 (11.1)				
Age						
≤50	371	31 (8.4)	1.00		1.00	
>50	487	64 (13.1)	1.69 (1.10 - 2.59)	0.017	1.45 (0.93-2.26)	0.102
Sex						
Female	362	26 (7.2)	1.00		1.00	
Male	496	69 (13.9)	2.07 (1.32 - 3.25)	0.002	1.52 (0.95 - 2.41)	0.079
Regional/distant metastasis						
No	709	51 (7.2)	1.00		1.00	
Yes	149	44 (29.5)	4.78 (3.19 - 7.15)	<0.001	3.24 (2.12-4.95)	<0.001
Breslow's tumor thickness (mm)						
≤1	347	7 (2.0)	1.00		1.00	
>1	511	88 (17.2)	9.17 (4.25 - 19.8)	<0.001	4.29 (1.78-10.30)	0.001
Ulceration						
No	681	48 (7.1)	1.00		1.00	
Yes	155	43 (27.7)	4.91 (3.25 - 7.42)	<0.001	2.57 (1.67-3.95)	<0.001
Missing	22					
Mitotic rate (mitoses/mm^2)						
<1	275	9 (3.3)	1.00		1.00	
≥1	583	86 (14.8)	4.67 (2.35 - 9.29)	<0.001	1.86 (0.92-3.80)	0.086
**Harvard**	409	48 (11.7)				
Age						
≤50	72	3 (4.2)	1.00		1.00	
>50	337	45 (13.4)	1.04 (1.01 –1.08)	0.003	1.05 (1.01-1.08)	0.004
Sex						
Female	271	31 (11.4)	1.00		1.00	
Male	138	17 (12.3)	1.16 (0.64 –2.10)	0.622	0.96 (0.52-1.76)	0.882

MSS: melanoma-specific survival; CM: cutaneous melanoma; SNP: single nucleotide polymorphism, HR: hazards ratio; CI: confidence interval; MDACC, MD Anderson Cancer Center.
*Multivariate Cox regression analyses were adjusted for all factors listed in this table.

Supplementary Table 2: Potential functional SNPs associated with MSS with FDR < 0.20 in the MDACC study

SNP	Gene	Chr.	Position	Location	MAF	A1/ A2	HR (95%CI)	*P*	FDR	SNPinfo[a]	RegulomeDB[b]
rs6707820	*NCK2*	2	106425713	Intron	0.13	C/T	2.19 (1.48-3.25)	9.53E-05	0.162	--	4
rs67594123	*NCK2*	2	106426378	Intron	0.11	G/C	2.38 (1.55-3.65)	7.86E-05	0.158	--	3a
rs7566776	*NCK2*	2	106427459	Intron	0.11	C/T	2.38 (1.55-3.66)	7.24E-05	0.158	--	--
rs58597816	*NCK2*	2	106428191	Intron	0.11	G/A	2.38 (1.55-3.66)	7.24E-05	0.158	--	5
rs2889602	*NCK2*	2	106429077	Intron	0.11	G/C	2.38 (1.55-3.66)	7.24E-05	0.158	--	4
rs2645768	*COL6A3*	2	238268454	Intron	0.20	A/C	1.91 (1.41-2.57)	2.40E-05	0.158	--	5
rs7597795	*COL6A3*	2	238270726	Intron	0.16	A/G	1.94 (1.40-2.68)	6.07E-05	0.158	--	5
rs7598394	*COL6A3*	2	238271229	Intron	0.16	A/G	1.93 (1.40-2.67)	6.53E-05	0.158	--	5
rs3773722	*PRKCD*	3	53221987	Intron	0.21	A/G	1.92 (1.39-2.63)	6.46E-05	0.158	--	6
rs2306574	*PRKCD*	3	53222761	Exon	0.21	T/C	1.92 (1.39-2.63)	6.46E-05	0.158	splicing	5
rs57742023	*PRKCD*	3	53226997	3' downstream	0.21	G/A	1.92 (1.40-2.64)	5.56E-05	0.158	--	4
rs13301426	*COL5A1*	9	137677921	Intron	0.08	C/T	2.36 (1.53-3.64)	9.54E-05	0.162	--	5
rs35128944	*COL5A1*	9	137679591	Intron	0.06	C/T	2.65 (1.65-4.25)	5.81E-05	0.158	--	5

SNP: single nucleotide polymorphism; MAF: minor allele frequency; MSS: melanoma-specific survival; FDR: false discovery rate; MDACC, MD Anderson Cancer Center; HR: hazards ratio; CI: confidence interval; A1/A2: reference allele/ effect allele.

[a] http://snpinfo.niehs.nih.gov.

[b] http://www.regulomedb.org.

Supplementary Table 3: Predictors of MSS obtained from stepwise Cox regression analysis in the MDACC study

Parameter[a]	Category[b]	Frequency	*P*	HR (95% CI)
rs13301426	CC/CT/TT	721/133/4	<0.001	2.27 (1.47-3.51)
rs2306574	TT/TC/CC	534/283/41	0.001	1.79 (1.29-2.49)
rs6707820	CC/CT/TT	644/204/10	0.007	1.74 (1.16-2.59)
rs2645768	AA/AC/CC	556/268/34	<0.001	1.88 (1.37-2.57)
age	≤50/>50	371/487	0.078	1.51 (0.96-2.39)
sex	Female/male	362/496	0.061	1.57 (0.98-2.51)
Regional/distant metastasis	No/Yes	709/149	<0.001	3.85 (2.51-5.92)
Breslow thickness (mm)	≤1/>1	347/511	<0.001	1.18 (1.11-1.25)
Ulceration	No/Yes	681/155	<0.001	2.44 (1.57-3.78)
Mitotic rate (mitoses/ mm^2)	≤1/>1	278/583	0.005	2.82 (1.37-5.81)

MSS, melanoma; MDACC, MD Anderson Cancer Center; HR, hazards ratio; CI, confidence interval.

[a]Stepwise Cox regression analysis included age, sex, regional/distant metastasis, Breslow thickness, ulceration, mitotic rate and four SNPs (*COL5A1* rs13301426, *PRKCD* rs2306574, *NCK2* rs6707820, and *COL6A3* rs2645768).

[b]The "category/" was used as the reference.

Supplementary Table 4: Association of four identified SNPs with overall survival of patients of European ancestry in the TCGA database

SNP	CHR	Position	Allele[a]	Gene	N	MAF	HR (95%CI)[b]	*P*[b]
rs6707820	2	106425713	C>T	*NCK2*	287	0.01	1.68 (0.52-5.41)	0.388
rs2645768	2	238268454	A>C	*COL6A3*	287	0.18	1.02 (0.72-1.44)	0.913
rs2306574	3	53222761	T>C	*PRKCD*	288	0.22	1.14 (0.88-1.49)	0.317
rs13301426	9	137677921	C>T	*COL5A1*	288	0.09	0.96 (0.62-1.48)	0.852

Abbreviation: CHR=chromosome; HR = hazards ratio; CI= confidence interval.
[a]Reference allele > effect allele.
[b]Adjusted for age, sex and stage.
